# DAPK1 loss triggers tumor invasion in colorectal tumor cells

**DOI:** 10.1038/s41419-019-2122-z

**Published:** 2019-11-26

**Authors:** Sara Steinmann, Philipp Kunze, Chuanpit Hampel, Markus Eckstein, Jesper Bertram Bramsen, Julienne K. Muenzner, Birgitta Carlé, Benardina Ndreshkjana, Stephan Kemenes, Pierluigi Gasparini, Oliver Friedrich, Claus Andersen, Carol Geppert, Shengbao Wang, Ilker Eyupoglu, Tobias Bäuerle, Arndt Hartmann, Regine Schneider-Stock

**Affiliations:** 1 Experimental Tumor Pathology, University Hospital Erlangen, Friedrich-Alexander University Erlangen-Nuremberg, Universitaetsstrasse 22, 91054 Erlangen, Germany; 2 Institute of Pathology, University Hospital Erlangen, Friedrich-Alexander University Erlangen-Nuremberg, Krankenhausstr. 8-10, 91054 Erlangen, Germany; 3 Experimental Trauma Surgery, Department of Trauma Surgery, University Regensburg Medical Center, Regensburg, Germany; 4 Department of Molecular Medicine, Aarhus University Hospital, Palle Juul-Jensens Boulevard 99, 8200 Aarhus N, Denmark; 5 Institute of Biotechnology, Friedrich-Alexander University Erlangen-Nuremberg, Paul-Gordan-Str. 3, 91052 Erlangen, Germany; 6 Department of Cancer Biology and Genetics, College of Medicine, The Ohio State University Comprehensive Cancer Center, Columbus, OH USA; 7 Department of Neurosurgery, University Hospital Erlangen-Nuremberg, Schwabachanlage 6, 91054 Erlangen, Germany; 8 Department of Radiology, University Hospital Erlangen-Nuremberg, Maximiliansplatz 1, 91054 Erlangen, Germany

**Keywords:** Colon cancer, Cell invasion, Cancer models

## Abstract

Colorectal cancer (CRC) is one of the leading cancer-related causes of death worldwide. Despite the improvement of surgical and chemotherapeutic treatments, as of yet, the disease has not been overcome due to metastasis to distant organs. Hence, it is of great relevance to understand the mechanisms responsible for metastasis initiation and progression and to identify novel metastatic markers for a higher chance of preventing the metastatic disease. The Death-associated protein kinase 1 (DAPK1), recently, has been shown to be a potential candidate for regulating metastasis in CRC. Hence, the aim of the study was to investigate the impact of DAPK1 protein on CRC aggressiveness. Using CRISPR/Cas9 technology, we generated DAPK1-deficient HCT116 monoclonal cell lines and characterized their knockout phenotype in vitro and in vivo. We show that loss of DAPK1 implemented changes in growth pattern and enhanced tumor budding in vivo in the chorioallantoic membrane (CAM) model. Further, we observed more tumor cell dissemination into chicken embryo organs and increased invasion capacity using rat brain 3D in vitro model. The novel identified DAPK1-loss gene expression signature showed a stroma typical pattern and was associated with a gained ability for remodeling the extracellular matrix. Finally, we suggest the DAPK1-ERK1 signaling axis being involved in metastatic progression of CRC. Our results highlight DAPK1 as an anti-metastatic player in CRC and suggest DAPK1 as a potential predictive biomarker for this cancer type.

## Introduction

Colorectal cancer (CRC) is the fourth most frequently diagnosed malignancy worldwide. Approximately 8.5% of patients with CRC die from their cancer^1^. Development of metastasis predominantly in liver and lung is the fatal leading cause of death and a serious obstacle of curing CRC. Recently, it has been shown that Death-associated protein kinase 1 (DAPK1) might be a potential candidate regulating metastasis in CRC^2–4^. DAPK1 as an actin filament-associated calcium/calmodulin-regulated, stress-responsive serine/threonine kinase was reported to be an extremely pleiotropic molecule due to its unique multi-domain structure^5–9^. Chen et al.^3^ have postulated that three main action modes for metastasis suppression by DAPK1 might exist: the increase in susceptibility of tumor cells to apoptotic signals, inhibitory role on integrin-mediated cell adhesion and migration and modulation of tumor microenvironment. Several reports have shown that primary tumors of patients with metastases (pM1) show decreased DAPK1 protein expression in comparison to primary tumors without metastases (pM0)^3,4^. Recently we have reported that the loss of anti-migratory function of DAPK1 could be one of the reasons for tumor cell dissemination^4^. In this regard it was not surprising that DAPK1 was nearly lost in tumor buds at the invasion front of CRC. Tumor buds are defined as single cells or small clusters of up to four cells^4^ and their evaluation at the stroma border of colorectal tumors is supposed to be indicative for tumor aggressiveness^4^. Moreover, tumor budding seems to be an independent and a reliable predictor of local lymph node metastasis, tumor recurrence and survival^10^. Since DAPK1 is lost in this aggressive cell subpopulation at the tumor invasion front we suggest a tumor suppressor role for DAPK1. Interestingly, DAPK1 was found to be involved in the cross talk of tumor cells with macrophages of the stroma environment^11^. At the onset of cancer, loss of DAPK1 may provide selective advantage for hyperproliferative tumor cells of evading the p53-dependent apoptotic checkpoint during transformation^12^. Indeed, DAPK1 loss has been shown in a high number of T1 colorectal tumors^13^. At later stages of tumorigenesis and during metastasis, tumor cells seem to benefit from loss of DAPK1 resulting in reduced sensitivity to detachment from extracellular matrix (ECM)^14^. So far, the molecular mechanisms behind metastasis in CRC and especially the role of DAPK1 in this process remain little understood.

To mirror-image DAPK1 loss in tumor buds and to shed more light into the role of DAPK1 in CRC aggressiveness we designed unique CRISPR/Cas9 DAPK1 knockout (ko) cell lines and characterized the knockout phenotype in vitro and in vivo. Although we observed a definite variation in functional readouts for the different clones, we were able to extract a uniform DAPK1-stroma specific gene expression pattern. In the chorioallantoic membrane (CAM) model, we showed an increased tumor budding and metastatic potential when DAPK1 was lost in tumor cells supporting its role as a tumor suppressor. We propose the DAPK1-ERK axis to be involved in metastasis suppression. Loss of adhesion molecule ICAM1, gain in TACSTD2 expression, and increased tumor cell–ECM interaction under DAPK1 loss seem to be central events in this process.

## Material and methods

### Cell lines, cell culture, and ERK1/2 inhibition

The human colorectal tumor cell lines HCT116, DLD1, SW480, SW620, SW837, and HT29 were obtained from ATCC. HCT116 cells line was used as in vitro model due to wildtype Tp53 status compared to other CRC cell lines (Supplementary Table 1). HCT116 and HCT116-derived monoclonal DAPK1 ko clones 7/6, 10/8 and 21/9 were maintained in RPMI 1640 medium supplemented with 10% (v/v) fetal bovine serum (FBS), 1% penicillin (100 U/ml) and streptomycin (100 µg/ml) (all from PAN Biotech, Aidenbach, Germany) and cultured in a humidified atmosphere of 5% CO_2_ at 37 °C. For ERK1/2 inactivation, cells were treated with 10 and 20 µM FR180204 (Selleckchem, Munich, Germany) for 48 h. All cell lines were genotyped using Multiplex Cell Authentication by Multiplexion (Heidelberg, Germany) as described recently^15^. Mycoplasma-free status has been verified for all cell lines.

### Establishment of DAPK1 ko cell lines

HCT116 cells were genome-edited using the CRISPR/Cas9 technology^16^. CRISPR/Cas9 vector pX330 was a gift from Feng Zhang (plasmid #42230, Addgene, Teddington, UK). The antibiotic selection vector pBABE-puro (Addgene plasmid #1764) was kindly provided by Professor Jeffrey Parvin (OSU, Columbus OH, USA). For CRISPR/Cas9-mediated gene ko, single guide RNAs (sgRNA) targeting exon 3 of human *DAPK1* (NM_004938; ENSG00000196730; sgRNA1: nt 611-629, sgRNA2: nt 615–634; kinase domain*)* were designed using a common CRISPR design tool (https://benchling.com/academic; Supplementary Fig. 1a). After annealing, the 20 nt targeting sgRNA (Supplementary Fig. 1b) were introduced into pX330 at its *BbsI* site. For transient transfection, 0.3 × 10^6^ cells per 6 well were seeded and cultured for approximately 24 h until 70–80% of confluency. 1.25 µg of pX330-DAPK1-sgRNA1 or pX330-DAPK1-sgRNA2 and 1.25 µg of pBABE-puro (plasmid #1764, Addgene, Teddington, UK)^17^ for antibiotic selection were transiently co-transfected into adherent HCT116 cells using Lipofectamine 2000 (Life Technologies/Thermo Fisher Scientific, Waltham, MA, USA) according to the manufacturer’s instructions. After 24 h transfected cells were maintained in culture medium containing 1.5 mg/ml puromycin for 19 days for positive selection. For isolation of monoclonal cell populations, surviving cells were harvested and seeded as limiting dilution (100 µl of a 4–5 cells/ml solution per 96 well). Single-cell colonies were expanded for DNA- and protein extraction and cryopreservation. Each clone was genotyped by Sanger sequencing (Seqlab, Germany) of PCR-amplified gDNA (sense: 5′- TCA ATC CCT CGT TTT TCA GG -3′, anti-sense: 5′- CCA ATT CCT GAT CCC TCT CTC -3′) using the forward primer 5′- CCA CAT CCT CAC TCA AAT CCT -3′.

### Nuclear/cytoplasmic fractionation of proteins

Sub-cellular fractions of the HCT116, HCT 7/6, and HCT 21/9 cells were prepared using REAP cell fractionation method^18^. Briefly, cell pellets were resuspended in 500 μl of ice-cold 0.1% NP40 (Calbiochem, CA, USA) in PBS, triturated five times using a p1000 micropipette and centrifuged for 10 s in 1.5 ml micro-centrifuge tubes. The supernatants were transferred to the new tubes and kept on ice (this is the cytoplasmic fraction). The pellets were washed with 1 ml of ice-cold 0.1% NP40-PBS lysis buffer, centrifuged for 10 s, and the supernatants were discarded. The remaining pellet was dissolved in 100 µl 0.1% NP40-PBS lysis buffer (this is the nuclear fraction). All lysates were analyzed by Western Bloting.

### Western Blotting analysis

Western Blotting was performed as previously described^4^. Briefly whole cell lysates were prepared in urea lysis buffer (4 M urea, 0.5% SDS, 62.5 mM Tris, pH 6.8) supplemented with 1% Protease inhibitor cocktail (Merck Millipore, Darmstadt, Germany) and 1 mM phenylmethylsulfonylfluorid (Roth, Karlsruhe, Germany). Sodium dodecyl sulfate polyacrylamide (PAA) Gel Electrophoresis (SDS-PAGE; 7.5–12% of PAA) was performed with 30–60 µg protein per sample and proteins were transferred onto nitrocellulose membranes (Whatman, Little Chalfont, UK) overnight. After blocking membranes were incubated with primary antibodies at 4 °C overnight and then horseradish-peroxidase (HRP)-conjugated secondary antibodies anti-mouse and anti-rabbit (1:10 000; Thermo Fisher Scientific, Waltham, MA, USA) were added for 1 h at RT. Chemiluminescence images were captured using the Gene Gnome chemiluminescence developer (Syngene, Bangalore, India). The primary antibodies were: anti-Cofilin (1:1000, sc-33779), -phospho-Cofilin^Ser3^ (1:500, sc-12912-R; both from Santa Cruz, Dallas, TX, USA), -DAPK1 (1:150, 610291; BD Biosciences, Heidelberg, Germany), -DAPK2 (1:250, PA141305; Life Technologies/Thermo Fisher Scientific, Waltham, MA, USA), -DRAK1 (1:500, PA5–21849), -DRAK2 (1:500, PA1-41308; both from Thermo Fisher Scientific, Waltham, MA, USA), h*SpCas9* (1:1000, C152002203; Diagenode, Seraing, Belgium), CD133 (1:250, 130-092-395; Miltenyi Biotec GmbH, Bergisch Gladbach, Germany), Lamin A + C (1:4000, AB108922); Abcam, Berlin Germany) -ERK1/2 (1:1000, 9102), pERK1/2 (1:1 000, 9101), -ICAM1 (1:250, 4915), -DAPK3 (1:1000, 2928), -CD44 (1:1000, 3570), -Vimentin (1:1000, 5741), -E-Cadherin (1:1000, 3195), p-MLC (1:500, 3671), and -TACSTD2 (1:1 000, 90540); all from Cell Signaling, Frankfurt am Main, Germany), Western Blot bands were quantified by densitometric analysis using ImageJ (National Institutes of Health; Bethesda, MD, USA). HRP-conjugated anti-GAPDH (1:75 000, MAB5476; Abnova, Aachen, Germany) served as loading control for protein normalization. Experiments were performed at least two times.

### WST-8-based cell proliferation assay

Proliferation rate was determined using the colorimetric Cell Counting Kit-8 (CCK-8, Dojindo, Munich, Germany) according to the manufacturer’s instructions. Briefly, cells (10 × 10^4^ cells/well) were seeded in a 96 well flat-bottom microplate and cultured in 200 µl of culture medium at 37 °C and 5% CO_2_ overnight to allow adherence and further cultured for 0, 3, 6, 24 and 48 h. After given time points, old medium was replaced by 100 µl fresh medium supplemented with 1% WST-8 reagent and incubated for further 2 h at 37 °C. Thereafter, 100 µl supernatant was transferred into a new 96 well and the absorbance was measured at 450 nm using the multilabel reader VICTOR^TM^ X3 (Perkin Elmer, Rodgau, Germany). Results are presented as mean ± SEM. A value of *P* < 0.05 was considered to be statistically significant.

### Chicken CAM assay

Fertilized, specific pathogen-free (SPF) chicken eggs (VALO Biomedia, Osterholz-Scharmbeck, Germany) were maintained at 37 °C and 80% constant humidity. On day 8, a window of 1.5–2.0 cm diameter was cut in the shell at the more rounded pole of the egg and sealed with tape (Durapore silk tape, 3 M). The next day, 1.0 × 10^6^ human tumor cells per pellet were embedded in growth factor-reduced matrigel (Corning, Wiesbaden, Germany) serving as matrix and were transplanted onto the CAM. The window was sealed again and eggs were incubated for additional 5 days. Tumor growth was monitored over time using a light microscope (×10, SU 1071 Traveler). Tumors were sampled with the surrounding CAM on day 5, fixed in 4% formaldehyde, paraffin-embedded and cut into 3–5 µm sections for immunohistochemical evaluation. For gene expression and protein expression analysis, fresh tumors were cryopreserved in liquid nitrogen and stored at −80 °C for long-term storage (44). For optical imaging, tumor cells were stained with CytoPainter Cell Proliferation Staining Reagent—Deep Red Fluorescence (ab176736, Abcam, Cambridge, UK) as described previously^19^.

### Immunohistochemistry

Serial sections (3–5 µm) of 4% formalin-fixed and paraffin-embedded CAM tumors were deparaffinized with xylene and rehydrated in graded alcohol. Validated protocols established for the clinical routine were applied for hematoxylin-eosin (HE), phospho-Histone 3 (pHH3, Cell Signaling, Frankfurt am Main, Germany), pan-Cytokeratin (pan-CK, Thermo Fisher Scientific, Waltham, MA, USA) staining. Anti-ICAM1 and anti-phospho-ERK1/2^Thr202/Tyr204^ (both from Cell Signaling, Frankfurt am Main, Germany) and anti-TACSTD2 (Abcam, Cambridge, UK) stainings were performed as follows: for antigen retrieval, slides were cooked in 1 mM Tris-EDTA buffer (pH 8.5) at 120 °C for 5 min. After peroxidase blocking (Dako/Agilent, Munich, Germany) for 5 min at RT, slides were incubated with primary antibodies anti-ICAM1 (1:50) and anti-phospho-ERK1/2^Thr202/Tyr204^ (1:50) for 30 min and with anti-TACSTD2 (1:2000) overnight at RT. ICAM1 and anti-phospho-ERK1/2^Thr202/Tyr204^ antibody binding was visualized by incubating the sections with the EnVision Detection System (Peroxidase/DAB, Dako) for 30 min at RT and subsequently with DAB substrate (Dako/Agilent, Munich, Germany) for 10 min at RT. For detection of TACSTD2 antibody binding, the ABC-kit from Vector Laboratieries (Burlingame, CA, USA) was used according to the manufacturer’s recommendations. Sections were counterstained with hematoxylin (Merck) for 1 min. Immunohistological stainings were brightfield imaged at a magnification of ×20 with the Olympus BX51 microscope and Olympus XC50 camera (Olympus Corporation, Hamburg, Germany) or were scanned using a Panoramic MIDI system (Camera type: CIS VCC-FC60FR19CL; objective: Plan-Apochromat; magnification: ×40; Camera adapter magnification: ×1, 3DHISTECH, Ludwigshafen, Germany) for digital analysis. Tumor budding^10^ of CAM tumors was determined using pan-CK stained sections and the high-power-field (HPF, ×40) method^20^. It was calculated as the average number of buds in 4–10 HPFs per sample. A respective budding score was defined as low-grade with an average of ≤1 and high-grade with an average of >1 buds per 4–10 HPFs. Vessel areas of CAM xenografts were determined in scans of HE stained sections. The tumor as well as the intratumoral vessels were annotated manually using the CaseViewer software (3DHISTECH, Ludwigshafen, Germany). The percentage of vessel area was calculated by relating the total area of intratumoral vessels to tumor area and expressed in percent (%). ICAM1, TACSTD2 and cytoplasmic and nuclear pERK immunoreactive scores (IRS) were determined by multiplying staining intensities (0–3) and the respective percentage of positive cells (0–100%, no positive cell −0; all cells positive – 100) according to Remmele and Stegner^1^. The product was divided by 10 to achieve an IRS between 0 and 27. The mitotic rate of cells in the CAM micro-tumors was determined using pHH3 stained sections. Quantification was conducted using the QuPath software (https://qupath.github.io/) semi-automatically analyzing 10 HPFs (×20 magnification) per section. The mitotic index per section is expressed as the mean number of positive mitotic figures in % of 10 HPFs.

### Ex ovo optical imaging

After performing the CAM assay using HCT116 and clone 21/9 cells, chicken embryos were sacrificed by decapitation on day 6 of tumor growth and were placed in an in vivo optical whole body imaging system (IVIS Spectrum CT, Perkin Elmer Rodgau, Germany). On the camera of the imaging system, images of the whole embryo were taken and overlayed with optical signals of deep red fluorescence labeled cells (CytoPainter Cell Proliferation Staining Reagent, Abcam, Cambridge, UK) which were acquired with the following parameters: Epi-illumination using an excitation filter for 605 nm and an emission filter for 660 nm, exposure time of 2 s and optical fields of view (FOV B) of 6.6 cm. After background correction (embryos with unstained tumor cells), the average radiant efficiency within the embryos was determined by selecting a rectangular ROI that covered the entire embryo. Embryos without tumor cell grafts served as baseline control.

### Organotypic brain slice culture

The brain slice culture system using genetically identical slices of 6-day-old rat (Wistar strain, Charles River) brains were utilized in order to study colorectal carcinoma cell invasion. HCT116, clone 7/6, clone 10/8 and clone 21/9 cells were labeled with a green fluorescent vital dye (Abcam, Cambridge, UK) for tracking invading cells and subsequently transplanted onto the brain slices (3.0 × 10^5^ cells per brain slice). For each cell line, brain precision-cut tissue slices (PCTS, 3 slices per 6 well) of 5 different rats were used. Slices of a sixth rat without tumor cell transplants served as negative control. Propidium iodide (PI) staining of all brain slices served as control for equal loss of vitality of the brain tissue (data not shown). The integrated fluorescence intensities of live invading tumor cells and PI signals were imaged (×2.0 magnification) and quantified after 1 and 3 days of tumor growth under a fluorescence microscope (Olympus IX71, Olympus, Hamburg, Germany). Data are reported as mean fluorescent intensities per brain slice.

### 3D-tumor spheroid-based migration assay

For spheroid formation, HCT116 cells and clone 7/6, clone 10/8 and clone 21/9 cells (2.0 × 10^3^ cells per 96 well) were seeded into a 96 well round-bottom plate. After 3 days, preformed multicellular spheroids were transferred into an uncoated 96-well flat-bottomed plate (one spheroid per well). Tumor cell migration/dissemination was monitored for 48 and 96 h and imaged using a light microscopy (Leica Dmi1 light microscope, ×10 HI Plan I objective, Leica, Munich, Germany). The area of migration was annotated using GIMP (GNU Image Manipulation Program, Version 2.8) and determined using a self-programmed macro using ImageJ 1.46r (National Institutes of Health) software as published before^19^. The assay was performed in technical replicates (*n* = 8–11). A comparable experiment was conducted using 1.0 × 10^3^ cells per spheroid (*n* = 10–12) showing analogous results.

### 3D-tumor spheroid-based invasion assay

Upon spheroid formation after 72 h, the invasive potential of tumor cells was analyzed by embedding the generated 3D-tumor spheroids in an artificial ECM. For this, compact spheroids were transferred into a 96-Well ULA round-bottom plate by pipetting 100 µL (using a 1250 µL pipette tip without filter on top of a 100 µL pipette tip) of medium containing one single spheroid into each well. In this way, each well contained only one spheroid. When the spheroids were already generated in a 96-well ULA round-bottom plate it was sufficient to gently remove 100 µL medium from each well. After thawing growth factor reduced Matrigel^®^ on ice, the 96-well plate was placed on ice for 5 min to cool the wells. In this way an early polymerization of the Matrigel® was prevented. Then, 100 µL Matrigel^®^ were dispensed carefully into each well and mixed with the remaining medium by slowly pipetting up and down several times without introducing air bubbles into the mixture. To ensure that the spheroids were in a central position, the plates were centrifuged at 300*g* for 3 min at 4 °C. The plates were then placed in an incubator at 37 °C for 1 h until the Matrigel^®^ had polymerized. Afterwards, 100 µL of complete growth medium was added to each well. The plates were incubated at 37 °C and 5% CO_2_ for up to 96 h. Tumor invasion was documented by taking pictures with an inverted light microscope in the brightfield channel at ×4 and ×10 magnification every 48 h.

### NanoString sample preparation and nCounter assay

Gene expression analysis was performed using the human nCounter^®^ PanCancer Progression Panel (NanoString Technologies, Hamburg, Germany). Total RNA was isolated from frozen cell pellets (48 h culture) by QIAzol-chloroform extraction followed by RNeasy Mini Kit (Qiagen, Hilden, Germany) preparation. Isolated RNA (100 ng) was processed through the NanoString nCounter Prep Station. Briefly, the hybridization reaction (3 µl Reporter CodeSet, 5 µl hybridization buffer, 2 µl Capture ProbeSet and 5 µl total RNA) containing 100 ng RNA (HCT116: *n* = 3; clone 7/6: *n* = 2; clone 10/8: *n* = 3; clone 21/9: *n* = 1) was conducted for 16 h at 65 °C. Subsequently, samples were processed according to the manufacturer’s instructions and signals of reporter probes were counted and tabulated using the nCounter Digital Analyzer (NanoString Technologies, Hamburg, Germany). Finally, housekeeping gene (geometric mean of 30 genes) normalization for quantitating gene expression levels, positive control normalization for background noise correction and data analysis was performed using the nSolver™ Analysis Software 3.0 (NanoString Technologies, Hamburg, Germany) and standard settings. The fold-change of counts was determined by averaging results per DAPK1 ko cell line and comparing them to average counts of HCT116 cells. Only significant (*P* < 0.05) fold changes >−1.5 or > + 1.5 fold were considered as differentially expressed in DAPK1 ko clones compared to the wildtype. Transcripts with a RNA count of <5 of all samples were excluded as they were considered as non-expressed. For statistical analysis of fold changes, *p* values of pairwise t-tests calculated by nSolver^TM^ were consulted. Expression profiling data are available online (GEO accession number: GSE130488).

### Quantitative real-time PCR

Total RNA from fresh-frozen cell line pellets (biological triplicates) and fresh xenograft tumors (3 tumors per cell line) was isolated using QIAzol and the RNAeasy Mini Kit (both from Qiagen, Hilden, Germany). For cDNA synthesis, 1 µg RNA and the QuantiTect^®^ Reverse Transkription Kit (Qiagen, Hilden, Germany) were used. Quantitative real-time PCR analysis was performed using the QuantiTect SYBR Green RT PCR (Qiagen, Hilden, Germany) on the thermocycler CFX96^TM^ and the Real-Time System C1000^TM^ (BioRad, Munich, Germany). Gene-specific primer pairs were: ß-2-microglobuline (b2m)-sense: 5′–GAC TTG TCT TTC AGC AAG GA-3′; b2m-antisense: 5′–ACA AAG TCA CAT GGT TCA CA-3′; icam1-sense: 5′–aag gtg acc gtg aat gtg ct-3′; icam1-antisense: 5′-cgc tgg cgg tta tag agg ta-3′; tacstd2-sense: 5′–TCC CCT TTC GGT CCA ACA AC-3′; tacstd2-antisense: 5′–AAA CGA TCC CGG GTT GTC AT-3′. For data analysis, raw counts were normalized to b2m gene (ΔCt = CT_target_ – CT_reference_). Fold induction to HCT116 cell line was calculated using the 2^−ΔΔCT^ method with ∆∆Ct = ∆CT_experimental_ − ∆CT_control_. Each sample was analyzed in triplicates (*n* = 9).

### Gene list enrichment analysis

Gene list enrichment analysis of significantly up- and down-regulated transcripts in DAPK1 ko clones vs. HCT116 wt were performed using the Enrichr tool^21^ and the integrated KEGG 2016 and REACTOME 2016 gene-set databases. Lists of either significantly upregulated (*n* = 22) or down-regulated transcripts (*n* = 12) were used as input and only gene sets with an adjusted *P* value < 0.05 were considered significantly enriched.

### Calculation of the stromal scores for RNAs deregulated by DAPK1 ko in HCT116 cells

A “stroma score” for RNAs with either “unaltered” (absolute fold change < 1.05), “down-regulated” (fold change < 1.25) or “upregulated” (fold change > 1.25) expression between DAPK1 ko clones and HCT116 wildtype cells were calculated to illustrate that up and down-regulated RNAs in DAPK1 ko clones have more frequent stromal cell origin (in CRC tumors) as compared to unaltered transcripts, which are primarily of epithelial origin, i.e. deregulated transcripts are likely to affect tumor microenvironment (“cell extrinsic”) rather than “cell intrinsic” processes. The “stroma scores” were calculated using a similar approach as in Bramsen et al.^22^ and is the fraction of mouse (i.e. stroma cell derived) vs. human (i.e. epithelial cancer cell derived) transcripts in human PDX tumors from mice evaluated by RNA sequencing by Isella et al.^23^ (used scores are listed in Supplementary Table 1). Hereby, a high and low stroma score indicate that transcripts are primarily of stroma cell (mouse) or cancer epithelial (human) origin in CRC tumors, respectively.

### Pre-ranked gene-set enrichment analysis (GSEA) of the DAPK1 ko expression signature

Pre-ranked GSEA was performed using the GSEA V3.0 software^24,25^ using standard settings and all gene sets included in the Molecular Signatures Database v6.2. As input we used expression fold change values (DAPK1 ko clones vs. HCT116) for the 770 transcripts profiled by the nCounter^®^ PanCancer Progression Panel (NanoString Technologies Hamburg, Germany). Gene sets were manually categorized into functional gene-set groups representing biological properties relevant to this paper using the following keywords for each category: ECM matrix (keywords: matrisome, ECM, extracellular collagen); EMT/invasion (keywords: mesenchymal, invasive, epithelial cell migration); integrin pathways (keyword: integrin); cell-substrate adhesion (keywords: matrix adhesion, substrate adhesion, focal adhesion); and healing/inflammation (keywords: wound, “inflam”); Cell–cell adhesion (keywords: cell cycle); chromosome organization (keywords: chromosome, chromatin). All gene sets containing the terms “positive regulation of”, “Negative regulation of”, “UP” and “DN” were omitted from the analysis.

### Nearest template prediction (NTP) classification of TCGA COREA samples and comparison to their CMS classification status

CRC samples from the TCGA project was classified into three categories “DAPK1 ko up sign.”, DAPK1 ko down-sign.”, and DAPK1 ko unaltered sign.” using the NTP module^26^ of GenePattern 2.0^27^. As input we used RNA sequencing profiles for 434 COREAD samples acquired from the UCSC XENA Public Data Hubs (https://xena.ucsc.edu/public-hubs/) as log2 (FPKM + 1) normalized RNA expression values for 20.530 genes. As classification templates we used the transcripts with either “unaltered” (absolute fold change < 1.05), “down-regulated” (fold change < −1.25) or “upregulated” (fold change > 1.25) expression between DAPK1 ko clones versus HCT116 wildtype cell line. NTP classifications were considered robust for samples with a Benjamini-Hochberg <0.05. Consensus molecular subtype calls for the COREAD samples were acquired provided by the Colorectal Cancer Subtyping Consortium^28^ (CRCSC; “CMS final network plus RFclassifier in non-consensus samples”. The analysis was restricted to CRC samples for which a CMS annotation was provided by the CRCSC.

### ECM cell adhesion assay

The adhesion capacity of DAPK1 ko cells to different protein substrates was analyzed using the ECM Cell Adhesion Array Kit, colorimetric (Merck, Millipore, Darmstadt, Germany) according to the manufacturer’s recommendations. Briefly, after rehydration of the plate strips, 0.5 × 10^6^ cells per 96 well were plated in triplicates per cell line and incubated for 1 h at 37 °C and 5% CO_2_. Thereafter, the supernatant was carefully removed, cells were washed with PBS and 100 µl per well of Cell Stain Solution was added. After an incubation of 5 min at RT, cells were washed with ddH_2_O and were solubilized for 15 min in 100 µl Extraction Buffer. The absorbance was then quantified at 570 nm in the multilabel reader VICTOR^TM^ X3 (Perkin Elmer, Rodgau, Germany). BSA-coated wells served as negative control. The data were expressed as BSA-corrected absorbances as percent increase of HCT116 cells.

### Second-harmonic generation (SHG) microscopy

We applied SHG microscopy in freshly harvested CAM tissues 3–5 days after tumor cell implantation using an upright multiphoton microscopy system (TriM-Scope II; LaVision BioTec GmbH, Bielefeld, Germany). A femtosecond Ti:sapphire laser was used for imaging at the excitation wavelength 800 nm. The CAM was brought into focus using an HC Fluotar L25x/0.95 W Visir water immersion objective (Leica, Munich, Germany).

### STRING bioinformatic analysis

For interpretation of the newly discovered DAPK1 ko gene expression pattern, protein-protein interactions and the interplay with the DAPK1-ERK2 axis were investigated using STRING interaction database (https://string-db.org/).

### Immunofluorescence staining

For immunofluorescence (IF) studies, 8.0 × 10^4^ cells grown in µ-slides 8 well (Ibidi, Martinsried, Germany) were fixed with 4% formaldehyde for 30 min at RT, permeabilized with IF buffer (PBS supplemented with 0.2% Triton X-100 and 0.05% Tween20) and then blocked with 1% BSA in IF buffer for 30 min at RT. After washing the cells with PBS-glycine (100 mM glycine in PBS) they were incubated with primary antibodies anti-DAPK1 (1:80, BD Biosciences, Heidelberg, Germany) or anti-phospho-ERK1/2^Thr202/Tyr204^ (1:80, Cell Signaling, Frankfurt am Main, Germany) diluted in IF buffer at 4 °C overnight. Cells were washed with PBS and incubated with appropriate fluorochrome-conjugated secondary antibodies AlexaFluor^®^ 488 (1:500, A-11029) or AlexaFluor^®^ 555 (1:500, A-21428) (both Thermo Fisher Scientific, Waltham, MA, USA) at RT for 2 h. Following another washing step, cells were counterstained with Hoechst 33342 (5 µg/ml, Sigma-Aldrich, Taufkirchen, Germany) for DNA staining and optionally with Alexa Fluor^®^ 568 Phalloidin (1:40 of ×40 stock, A12379; Thermo Fisher Scientific, Waltham, MA, USA) to visualize cytoskeletal F-actin. Representative images were captured using a fluorescence microscope Nikon Eclipse Ti using 100x oil immersion objective lens (both from Nikon, Düsseldorf, Germany). Confocal images for phospho-ERK1/2^Thr202/Tyr204^ were obtained using a confocal laser scanning microscopy system (LSMT-PMT Observer ZI, LSM 710, Carl Zeiss Inc, Oberkochen, Germany) with a ×63 oil objective. Images were background corrected using ImageJ analysis software.

### Transient siERK2 transfection experiment

To obtain an ERK2 knockdown, HCT116 and DAPK1 ko clone 7/6 and 21/9 cells were grown to 70% confluence in a 6 well culture plate and transfected with DharmaFECT reagent and 100 nM of siRNA (SMARTpool: ON-TARGETplus Human MAPK3 (ERK2) siRNA (both from Dharmacon, Lafayette CO, USA) according to the manufacturer’s instructions and incubated for 48 and 72 h. Transfection with non-targeting SMARTpool siRNA served as negative control. The knockdown efficiency was determined by Western Blotting. ERK2 knockdown experiments were repeated in two independent experiments and representative Western blots are shown.

### Statistical analysis

All statistical tests (using two-tailed Mann–Whitney test, one-way ANOVA multiple comparison, unpaired t-test, multiple t-test and Pearson’s correlation) were performed using Prism 7 (San Diego, California, USA). Differences were considered statistically significant according to values of two-tailed *P < 0.05, **P < 0.01, ***P < 0.001. Types of the tests are indicated in the figure legends.

## Results

### Evaluation of HCT116-derived CRISPR/Cas9-mediated DAPK1 ko clones

**Fig. 1:**
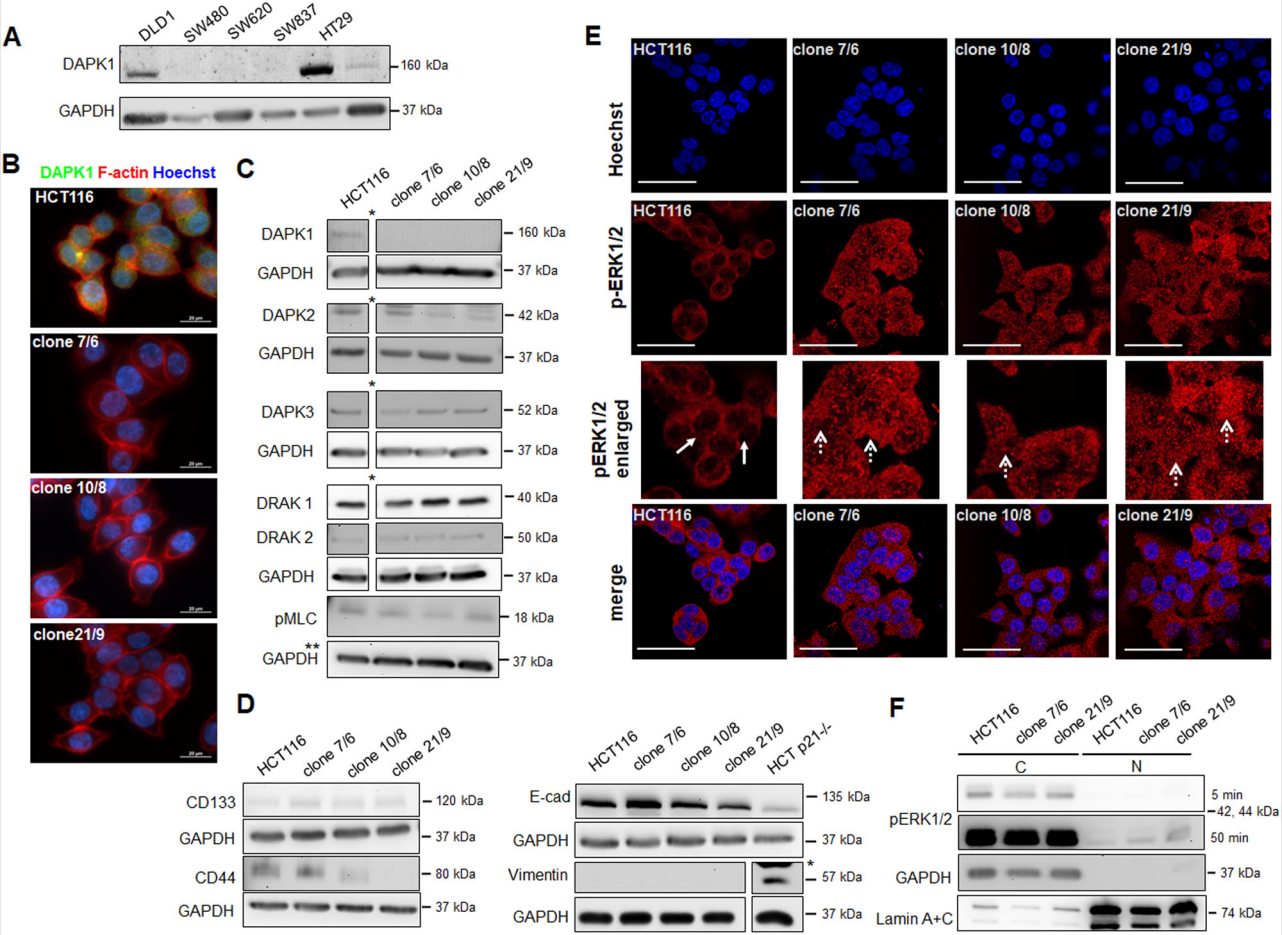
Validation of CRISPR/Cas9-mediated DAPK1 knockout in HCT116 colorectal cancer cells. **a** Western Blot of DAPK1 expression status in various colorectal cancer cell lines. Representative images of two independent experiments are shown. GAPDH served as loading control. **b** Immunofluorescence staining of DAPK1 (green) in parental HCT116 cells and DAPK1 ko clones. Cells were counterstained with phalloidin for F-actin (red) and nuclear Hoechst (blue). Fluorescence microscopy was performed using a x 100 oil immersion objective. Representative images of two independent experiments are shown. Scale bar = 20 µm. **c** Protein expression of DAPK1 family members DAPK1, DAPK2, DAPK3, DRAK1, DRAK2 and DAPK1 phosphorylation target pMLC in HCT116 wildtype cells and DAPK1 ko clones were detected by Western Blotting using specific primary antibodies. Representative images of two independent experiments are shown. * images were cropped here; all samples were analyzed on the same SDS-PAGE gel; ** GAPDH blot has been used twice see Fig. 5b, proteins have been loaded on the same membrane. **d** Western Blot analysis of stem cell markers (CD133, CD44) and EMT markers (epithelial marker: E-cad = E-cadherin, mesenchymal marker: Vimentin). Representative images of two independent experiments are shown.*images were cropped here; all samples were analyzed on the same SDS-PAGE gel. **e** Representative images of endogenous phospho-ERK1/2 (red) levels of immunostained HCT116 cells and DAPK1 ko clones examined by confocal immunofluorescence microscopy (63x; enlarged: cropped and zoomed in). Cells were nuclear counterstained with Hoechst (blue). Immunofluorescence was repeated in two independent experiments and representative images are shown. White arrows: empty nucleus; dashed arrow: nuclear expression of pERK1/2. Scale bar = 50 µm. **f** pERK1/2 expression analyzed by Western Blot in cytoplasmic (C) and nuclear (N) protein fractions. Representative images of two independent experiments are shown. GAPDH served as loading control in total and cytoplasmatic protein fractions. Lamin A/C was used for nuclear loading control.

Basal level of DAPK1 protein in the HCT116 cell line was determined using Western blot (Fig. 1a). HCT116 express low to moderate DAPK1 compared to DLD1 and HT29, which express high levels of DAPK1, and in comparison to SW480, SW620 and SW837, where DAPK1 levels were not detected (Fig. 1a). To mirror-image the loss of DAPK1 protein observed at tumor invasion front of colorectal tumors, we first established CRISPR/Cas9-driven DAPK1 ko HCT116 cell lines using two different single guide RNAs (sgRNA1 and 2) (Supplementary Fig. 1a, b). The presence of Cas9 protein, which serves as evidence for an active CRISPR/Cas9 system was shown in the lysate of HCT116 cells 24 h after transient transfection with sgRNA1 or sgRNA2 vectors by Western Blot whereas it was no longer detectable in the established monoclonal ko cell clones (Supplementary Fig. 1c). Sanger sequencing analysis revealed homozygeous and sgRNA-specific insertion mutations resulting in a reading frame shift (Supplementary Fig. 1d). Three monoclonal DAPK1 ko cell lines named clone 7/6, 10/8 and 21/9 were randomly chosen from a panel of several successfully generated subclones and the lack of endogenous DAPK1 protein was verified by immunofluorescence staining (Fig. 1b; Supplementary Fig. 2a) and Western Blotting (Fig. 1c). To exclude CRISPR/Cas9-caused off-target effects on other highly related DAPK family members, we determined the protein expression of DAPK2 (DRP-1), DAPK3 (ZIPK), DRAK1 and DRAK2 in Western Blotting (Fig. 1c). Steady state protein levels in HCT116 cells and in 10/8 and 21/9 revealed no ko effects on these molecules. The protein levels of pMLC, a well known DAPK1 target, were decreased in the DAPK1 ko clones (Fig. 1c). Regarding “stemness”, we found differences between the single clones with clone 7/6 had the highest expression of CD133 and CD44 markers and clone 21/9 did not express CD44 at all (Fig. 1d). No epithelial-to-mesenchymal transition (EMT) was observed in DAPK1 clones visualized by steady E-cadherin protein levels and lack of vimentin expression (Fig. 1d).

In confocal immunofluorescence images we showed that pERK1/2 in HCT116 cells is predominantly localized in the cytoplasm (Fig. 1e), whereas in the three DAPK1 ko clones pERK1/2 expression was remarkably increased in the nuclei of tumor cells in vitro (Fig. 1e). These findings were confirmed analyzing nuclear and cytoplasmatic protein fractions in Western Blot (Fig. 1f) indicating that the loss of DAPK1 is associated with nuclear pERK1/2 shuttling under endogeneous conditions.

The functional DAPK1 ko was verified in a 3D migration spheroid system. We evaluated the whole areas since the border of the cores were not well defined. At day 4 we confirmed an anti-migratory role of DAPK1 in all three clones (Supplementary Fig. 3a, b). Moreover, in agreement with our previous findings^29^ with DAPK1 as a pro-apoptotic player, the TNF-α induced phosphorylation of Cofilin was remarkably diminished in DAPK1 ko clones (Supplementary Fig. 3c). In summary, these findings strongly support the functional ko in our newly generated DAPK1 ko cell lines.

### In vivo growth pattern of DAPK1 ko clones

**Fig. 2:**
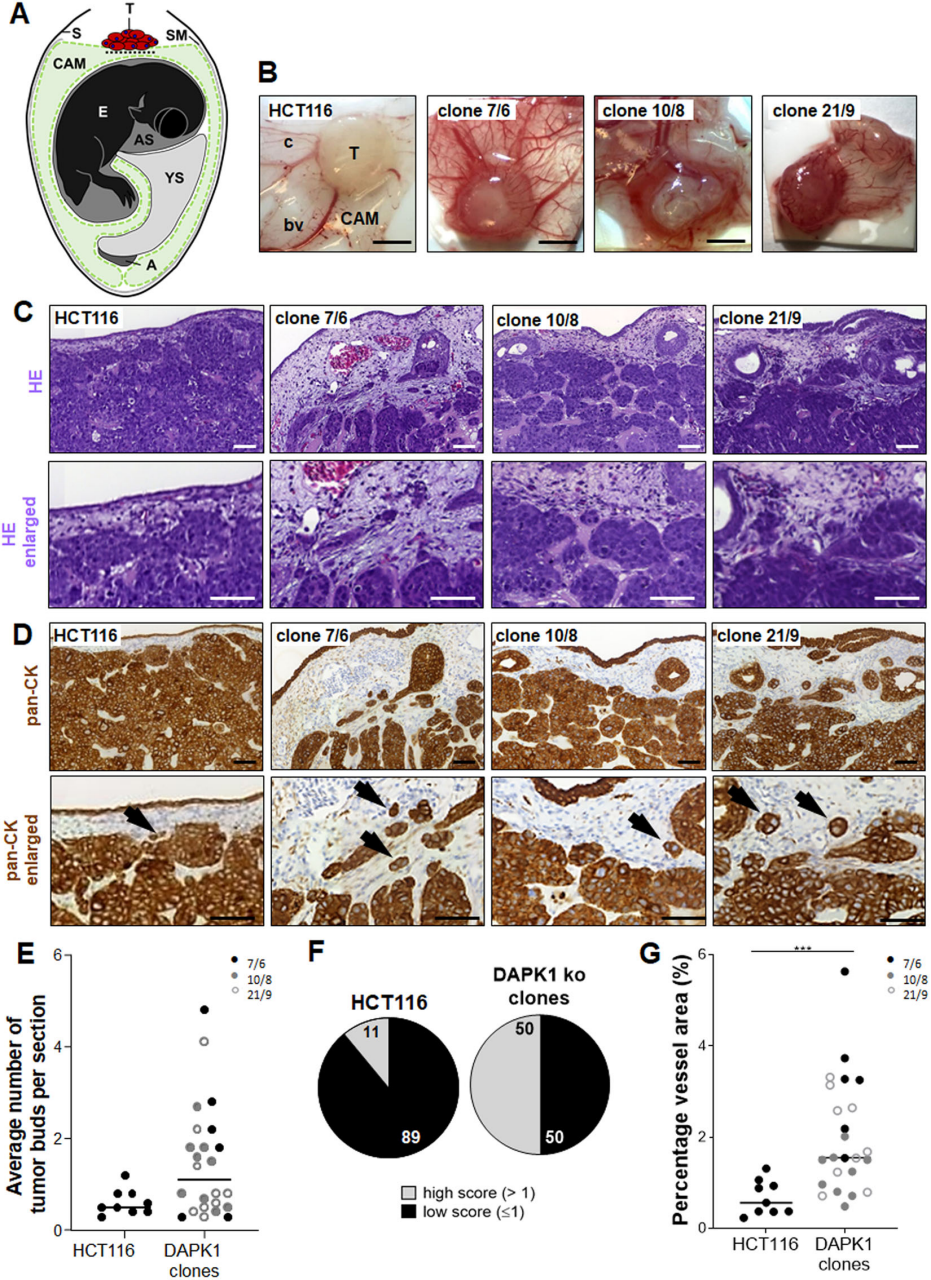
Tumor growth of HCT116 cells and DAPK1 ko clones in the chicken CAM in vivo model. **a** Scheme of a chicken CAM tumor assay. A albumen, AS amniotic sac, CAM chorioallantoic membrane, E chick embryo, S egg shell, SM shell membrane, T human tumor cells in Matrigel, YS yolk sac, dotted line invasion front of tumor cells investigated using pan-CK immunohistology. **b** Representative ex ovo images of HCT116 and DAPK1 ko clone derived xenograft tumors harvested 5 days post-implantation on the CAM of fertilized chicken eggs. C capillaries, T tumor, bv blood vessel, CAM chorioallantoic membrane. Scale bar = 3 mm. **c** Representative images of hematoxylin-eosin (HE) and **d** pan-cytokeratin (pan-CK) stained paraffin sections of CAM tumors (×20). Arrow head: tumor bud. Scale bar = 50 µm. **e** Average number of tumor buds (≤tumor 4 cells) counted on pan-CK stained sections across 4–10 high-power fields (HPFs; ×40). HCT116: *n* = 9; ko clones clone 7/6: *n* = 6; clone 10/8: *n* = 9; clone 21/9: *n* = 9; *P* = 0.06; Mann–Whitney test). **f** Pie chart diagram of high-score (≤1 mean bud number per up to 10 HPFs) and low-score (>1 buds) tumor budding. HCT116: *n* = 9 (*n*_low_ = 8; HCT116: *n*_high_ = 1); DAPK1 ko clones: *n* = 24 (*n*_low_ = 12; *n*_high_ = 12. **g** Percentage of intratumoral vessels counted on scans of HE stained sections expressed in %. HCT116: *n* = 9; DAPK1 ko clones: *n* = 24 (clone 7/6 *n* = 6; clone 10/8 *n* = 9; clone 21/9 *n* = 9). ***P ≤ 0.001 compared to HCT116 (Mann–Whitney test).

Next, we used the CAM assay to examine the role of DAPK1 loss for tumor growth. DAPK1 ko clones and parental HCT116 cells were transplanted onto the chicken CAM and were cultured in ovo for 5 days as schematically illustrated in Fig. 2a. Examples of CAM xenografts are shown in Fig. 2b. Conventional HE staining of HCT116-derived CAM tumors reflected the typical growth pattern of a microsatellite instable colorectal carcinoma with well-differentiated epithelial-like glandular structures and clear margins pushing back the chicken connective tissue (Fig. 2c). In contrast, DAPK1-deficient tumors showed a shift to loosely packed tumor masses and a highly infiltrative growth pattern intruding the CAM (Fig. 2c).

**Fig. 3:**
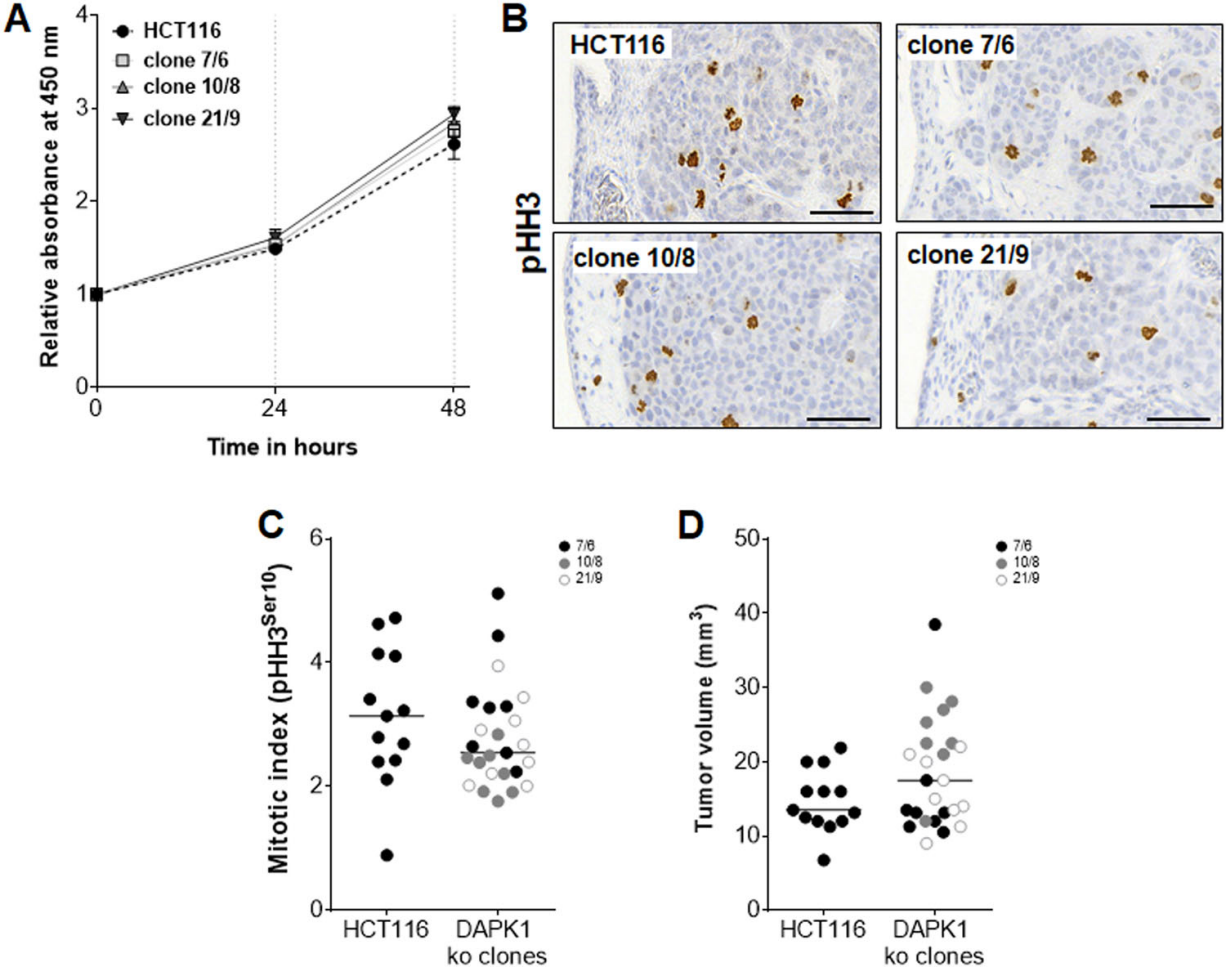
The proliferative capacity of HCT116 cells and DAPK1 ko clones in vitro and in vivo and CAM tumor volumes. **a** WST-8 assay of HCT116 cell and DAPK1 ko clones 7/6, 10/8 and 21/9 was conducted for quantification of the metabolic activity after pre-incubation of 0, 24 and 48 h and 2 h of WST-8 treatment. Absorbance was measured at 450 nm and presented as a relative growth curve with respect to time-point = 0 h. The mean of two independent experiments (*n* = 4 replicates per experiment) is shown, (*P* > 0.5; two-way ANOVA) **b** Immunohistochemical staining of mitoses in CAM tumor sections derived from HCT116 cells and DAPK1 ko clones using pHH3^Ser10^ specific primary antibody. Representative images are shown. Scale bar = 50 µm. **c** The mitotic rate of cells in CAM tumors was determined using pHH3 immunohistochemistry. Quantification of mitoses per 10 HPFs per section (×20) was performed with QuPath software (https://qupath.github.io/). The average number of mitoses per section is presented as dot. Medians of the data are presented as lines in the scatter plots. (HCT116: *n* = 13; clone 7/6: *n* = 8; clone 10/8: *n* = 8; clone 21/9: *n* = 9 (*P* = 0.1884; Mann–Whitney test). **d** Volumes of 5-day-old CAM xenograft tumors were measured and calculated as follows assuming an ellipsoid shape: *V*_Tumor_ = length × width × height × π/6 and presented as dots. Medians of the data are presented as lines in the scatter plots. HCT116: *n* = 13; clone 7/6: *n* = 8; clone 10/8: *n* = 8; clone 21/9: *n* = 9 (*P* = 0.1652; Mann–Whitney test).

Pan-CK staining of CAM xenografts was used to identify human CRC cells in the chicken mesodermal layer and to highlight tumor budding (Fig. 2d). The avian epithelial monolayers were also Pan-CK positive. Quantification of peritumoral budding, single cells or small clusters of up to four cells ahead of the invasive front^10^, revealed a trend of an increase in budding in DAPK1 ko tumors at day 5 (not significant, Fig. 2e). When evaluating a budding score with low-grade budding defined as an average of ≤1 and high-grade budding as >1 buds per 10 high-power-fields (HPFs; ×40), the DAPK1-dependent difference was remarkable. DAPK1 loss was associated with high-grade tumor budding (50% of evaluated tumors) demonstrating that every second HPF of DAPK1 negative tumors was given a high-grade score whereas 89% of all HCT116 wildtype tumors were low-grade budders (Fig. 2f). Since CAM assay is a classical in vivo model for angiogenesis, we analyzed neovascularization in HCT116 and DAPK1 ko tumors. DAPK1 ko tumors showed higher vessel area compared to HCT116 (Fig. 2g; Supplementary Fig. 4) indicating DAPK1 being an anti-angiogenic factor in CRC. Proliferation did not differ in a DAPK1-dependent manner in vitro (Fig. 3a) and in vivo when evaluating the pHH3 positive tumor cell population (Fig. 3b, c) in the CAM model which is in close agreement with the rather similar tumor volumes of CAM xenografts (Fig. 3d).

**Fig. 4:**
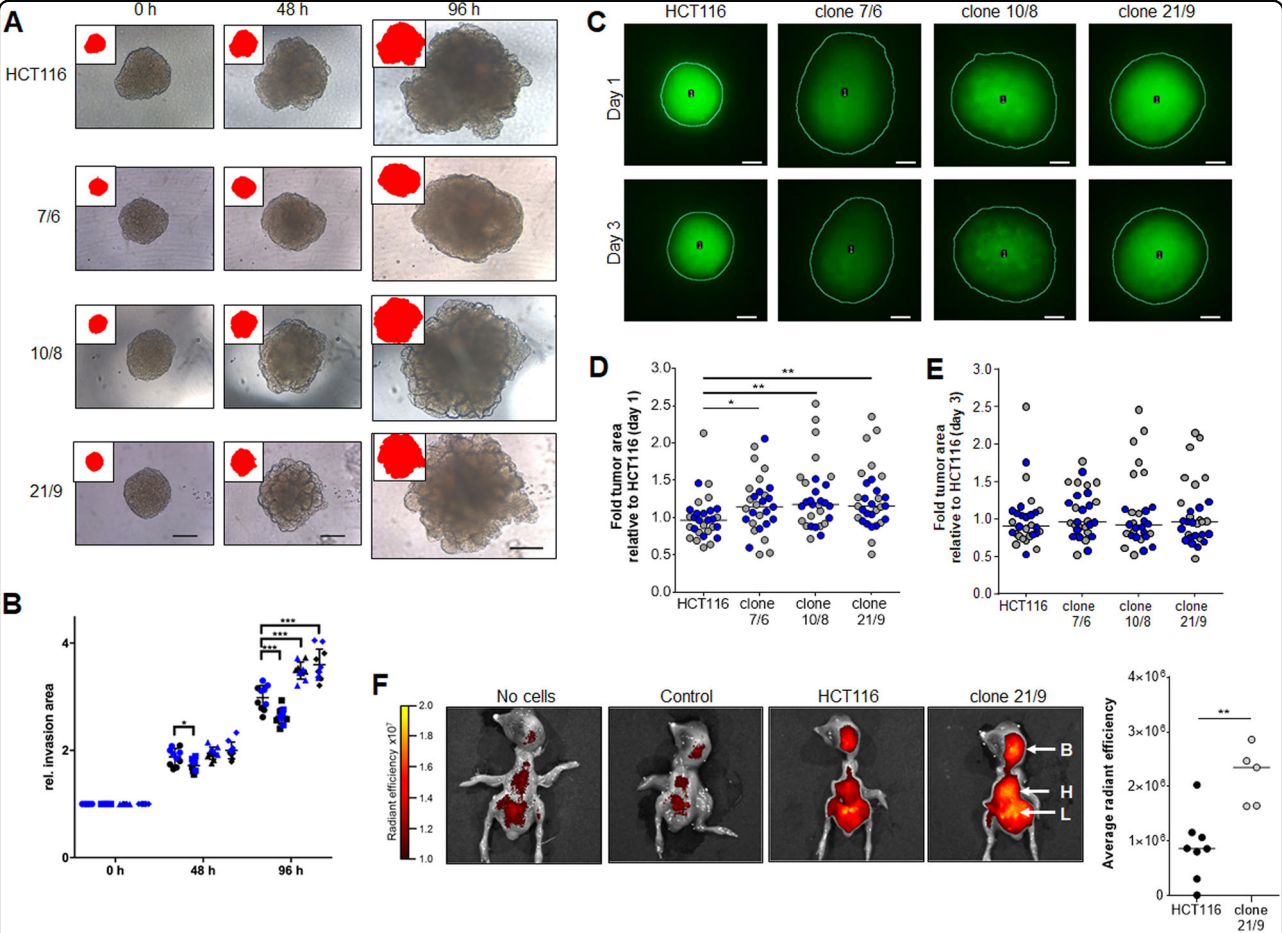
Enhanced metastatic capacity of DAPK1-deficient tumor cells. **a** Light microscopy images of embedded 3D spheroids taken after 0 h, 48 h and 96 h of incubation. The manual selection of the spheroid area (red) is displayed in the upper left corner of each image. Pictures were taken at ×10 magnification. Scale −200 μm. **b** Quantification of the invasive potential of n 3D spheroids from two independent experiments (1. experiment: displayed in black; 2. experiment: displayed in blue). All values represent means ± S.D. and statistical analysis was performed using two-way ANOVA followed by Dunnett’s multiple comparisons test (HCT116 (●): *n* = 5/5; clone 7/6 (■): *n* = 5/5; clone 10/8 (▲): *n* = 5/5); clone 21/9 (◆): *n* = 5/5). (*P ≤ 0.05 and ***P ≤ 0.001). **c** HCT116 and DAPK1 ko cells were implanted on living organotypic brain slices. Areas of tumor cell invasion one and three days after implantation were analyzed. Representative images (×2.0) of fluorescence intensities of two independent experiments are shown. Relative tumor invasion was detected by mean fluorescence intensities (green). **d** one day (*P < 0.0368, **P < 0.0028 compared to HCT116) and (**e**) three days after implantation and presented as fold-change to HCT116. Black/gray dots present the first (*n* = 15), blue dots the second (HCT116: *n* = 14; clone 7/6: *n* = 14; clone 10/8: *n* = 14; clone 21/9: *n* = 15) experiment. **f** Optical overlay of representative surface images and fluorescence signal of deep-red fluorescence labeled HCT116 and clone 21/9 cells in chicken embryos 6 days after tumor cell implantation. Embryos with unstained tumor cell implants served as background control. The color bar indicates the range of average radiant efficiency ×10^7^ from minimal (black) to maximal (yellow); Average radiant efficiency as detected by fluorescence imaging. B: brain; H: heart; L: lung." HCT116: *n* = 8; clone 21/9: *n* = 5. (**P < 0.0062; Mann–Whitney test).

For in vitro analysis of invasive potential of HCT116 and DAPK1 ko clones 7/6, 10/8 and 21/9 tumor cells, 3D-tumor spheroids were embedded in matrigel and cell penetration from the spheroid core into the environment was monitored over time (Fig. 4a). Even though none of the cell lines developed any invadopodia-like spikes, we observed outgrowth of compact protrusions in HCT116, clone 10/8 and 21/9 after 48 h and even more pronounced bulges after 96 h. Clone 7/6, however, failed to develop protrusions. Applying semi-automated quantification of the invasion area, we investigated that DAPK1 ko clones 10/8 and 21/9 exhibited significantly enhanced (***P < 0.001) invasive capacity compared to HCT116 after 96 h, while clone 7/6 revealed significantly smaller invasive areas after 48 h (*P < 0.05) and 96 h (**P < 0.01; Fig. 4b).

Next we confirmed the highly invasive behavior of DAPK1 ko tumor cells in another 3D ex vivo model, where tumor cells were applied and cultured on precision-cut tissue slices of rat brain (Fig. 4c). These brain slices at least partly reflect the complexity of an organotypic environment. Tracking green fluorochrome-labeled tumor cells, we found larger areas of invasion for DAPK1 ko clones one day after transplantation reflecting their increased invasive capability in a physiological 3D matrix in contrast to HCT116 wildtype cells (*P < 0.05; **P < 0.01; Fig. 4c, d). Although a high variability, significance levels were reached for all three ko clones at day one. However, at day three after transplantation, tumor areas reached nearly equal fluorescence intensities (Fig. 4e) suggesting that DAPK1 ko cells have a better early adaptation capability to the 3D environment. Notably, just like the CAM tumors (Fig. 2c), HCT116 cells generated dense tumor masses while DAPK1 ko clones formed more loosely packed and more spacious tumors as reflected by a more diffuse green fluorescence (Fig. 4c). PI staining of empty brain slices and slices with transplanted HCT116 and DAPK1 ko cells for quality control showed equal mean intensities of PI signal at day 1 and day 3 of the experiment (Supplementary Fig. 5a). The slight decrease in tumor area at day 3 might be caused by a loss of fluorescent dye due to proliferation of cells.

Furthermore, we examined if the increase in tumor budding was associated with an enhanced potential to form metastases in the chicken embryonic organs. Since DAPK1 loss is associated with anoikis resistance^30^, we hypothesized that DAPK1 ko tumor cells might have a survival benefit in the vascular system reflected by a higher number of disseminating tumor cells in the embryonic chicken organs. To test this hypothesis, pre-labeled HCT116 and DAPK1 ko clone 21/9 tumor cells were transplanted onto the CAM and whole bodies of freshly sacrificed chicken embryos were screened for optical signals using an intravital imaging system (IVIS Spectrum, Perkin Elmer; Fig. 4f, Supplementary Fig. 5b). Clone 21/9 seems to be representative since it showed more migration, more invasion and an elevated number of budding. Results exposed significantly higher average radiant efficiency (**P < 0.01) within animals loaded with clone 21/9-derived DAPK1 ko tumors (Fig. 4f) suggesting that DAPK1 ko cells showed more disseminating tumor cells, preferentially accumulating in the liver, heart, and brain of the chicken embryo.

### Identification of DAPK1 ko-related gene expression signature

**Fig. 5:**
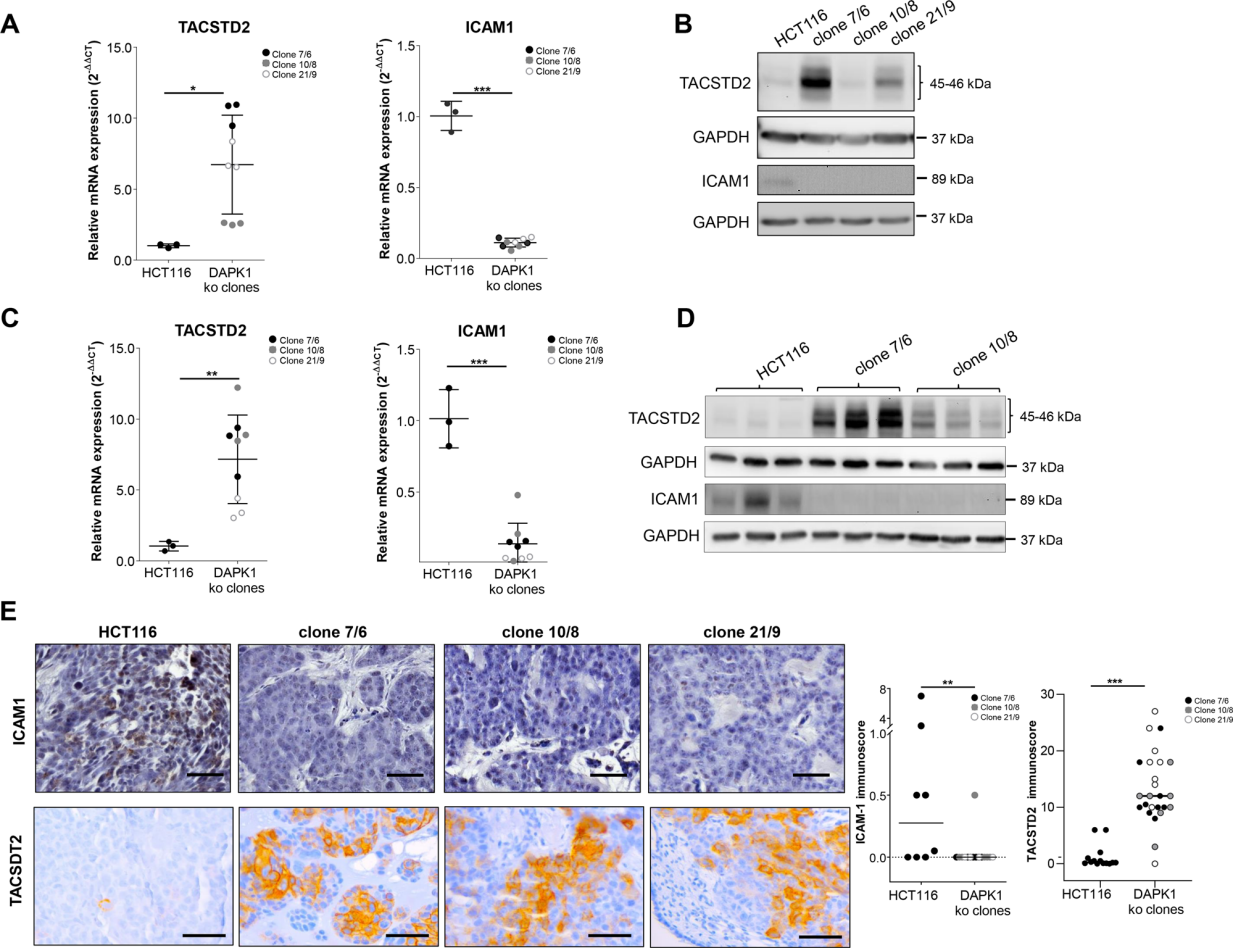
TACSTD2 increase and ICAM1 decrease of endogenous expression levels in DAPK1 ko clones. **a** qRT-PCR analysis of TACSTD2 (*P = 0.0204) and ICAM1 (***P < 0.001) gene expression in DAPK1 ko clones compared to wildtype HCT116 cells. B2M was used as reference gene. Three independent experiments per cell line are shown with one dot representing the mean of three technical replicates. Error bars represent mean ± SD. Unpaired t-tests were performed to compare qRT-PCR data. **b** Western Blot analysis of endogenous TACSTD2 and ICAM1 protein levels in HCT116 cells and DAPK1 ko clones. GAPDH served as loading control. Representative blots of two independent experiments are shown. **c** qRT-PCR of TACSTD2 (**P = 0.0082) and ICAM1 (***P < 0.001) gene expression in 5-day-old CAM micro-tumors. B2M was used as reference gene. Three independent experiments per cell line are shown with one dot representing the mean of three technical replicates. Error bars represent mean ± SD. Unpaired t-tests were performed to compare qRT-PCR data. **d** Western Blot analysis of TACSTD2 and ICAM1 expression in CAM tumor tissue (HCT116: 3 eggs, clone 7/6: 3 eggs, clone 10/8: 3 eggs). GAPDH served as loading control. **e** Representative images of ICAM1 and TACSTD2 immunohistochemistry of CAM micro-tumors (formalin-fixed and embedded in paraffin). Representative images are shown. Scale bar = 50 µm. ICAM1 immunoscores were obtained by multiplying staining intensity with percentage positivity divided by 10 (HCT116: n = 8; clone 7/6: 7, clone 10/8: 6, clone 21/9: 4). TACSTD2 immunoscores were obtained by multiplying staining intensity with percentage positivity (HCT116: *n* = 14; clone 7/6: *n* = 9; clone 10/8: *n* = 7; clone 21/9: *n* = 9). A Mann–Whitney test was performed to calculate significance between two groups (**P = 0.0037, ***P < 0.0001).

For determination of the DAPK1-dependent gene expression signature, we performed a NanoString-based gene expression analysis. Our panel covered genes which are involved in ECM remodeling, epithelial–mesenchymal transition (EMT), metastasis, and angiogenesis, some major hallmarks crucial for tumor progression. Parental HCT116 cells and each of the three DAPK1 ko clones revealed high similarity in their overall gene expression signature (Pearson’s *r* = 0.99, ***P < 0.0001; data not shown). When screening for genes similarly altered in all three DAPK1 ko cell lines 7/6, 10/8 and 21/9, a significant increase (>1.5 fold) in *IL11, TACSTD2, CLEC2B, P3H2, LAMA4, CCL5, SPDEF, CHRDL1, SERPINE1, COL5A1, HEG1, AGRN, TGFB2, COL7A1, GALNT7, PLAUR, CGN, ITGA1, NOTCH1, TBXA2R, LAMA5* and *TNS1* and decrease (>1.5 fold) in *JAG1, CALCRL, GPR124, SCG2, INHBE, CADM1, ACVRL1, COL6A3, FGF9, AGT, ANGPT2* and *ICAM1* were found (Table 1). Exemplarily, we selected TACSTD2 and ICAM1 as interesting candidates upon DAPK1 loss since they are majorly involved in ECM interaction and metastasis. We confirmed the endogenous *TACSTD2* up- and *ICAM1* downregulation on gene expression and protein levels using qPCR analysis (Fig. 5a) and Western Blotting (Fig. 5b) in all DAPK1 ko cell clones. The same effects on TACSTD2 and ICAM1 were confirmed in snap-frozen DAPK1 ko CAM tumors when analyzing mRNA expression (*P < 0.05; **P < 0.01; Fig. 5c) and protein levels using Western Blot (Fig. 5d). ICAM1 loss and TACSTD2 gain were also validated in vivo in DAPK1 ko CAM tumors by immunohistochemistry (**P < 0.01; Fig. 5e). Finally, we could show that TACSTD2 expression was dramatically enhanced in all DAPK1 ko clones 7/6, 10/8 and 21/9 (****P < 0.0001) compared to HCT116 in CAM xenografts (Fig. 5e).

**Table 1:** Fold change difference of mRNA expression of DAPK1 ko clones versus HCT116.

Reference sequence	Gene ID	Gene name	Average fold change (log2) ± SEM	*P*-value (*t*-test)
*A: upregulated genes*				
NM_000641.2	*IL11*	Interleukin-11	5.91 ± 0.76	<0.001
NM_002353.2	*TACSTD2*	Tumor-associated calcium signal transducer 2	5.13 ± 3.12	0.004
NM_005127.2	*CLEC2B*	C-type lectin domain family 2 member B	4.71 ± 7.14	0.016
NM_018192.2	*P3H2*	Prolyl 3-hydroxylase 2	2.78 ± 0.64	0.007
NM_001105209.1	*LAMA4*	Laminin, alpha 4	2.31 ± 0.47	0.031
NM_002985.2	*CCL5*	Chemokine (C-C motif) ligand 5	2.25 ± 0.33	0.05
NM_012391.1	*SPDEF*	SAM pointed domain containing ets transcription factor	2.22 ± 0.43	0.031
NM_001143981.1	*CHRDL1*	Chordin-like 1	2.22 ± 0.36	0.011
NM_001165413.1	*SERPINE1*	Serpin peptidase inhibitor, clade E (nexin, plasminogen activator inhibitor type 1), member 1	2.07 ± 0.34	0.001
NM_000093.3	*COL5A1*	Collagen, type V, alpha 1	1.95 ± 0.96	0.024
NM_020733.1	*HEG1*	Protein HEG homolog 1	1.78 ± 0.28	0.003
NM_198576.2	*AGRN*	Agrin	1.75 ± 0.16	<0.001
NM_003238.2	*TGFB2*	Transforming growth factor, beta 2	1.73 ± 0.36	0.012
NM_000094.2	*COL7A1*	Collagen, type VII, alpha 1	1.63 ± 0.85	0.037
NM_017423.2	*GALNT7*	UDP-N-acetyl-alpha-D-galactosamine:polypeptide N-acetylgalactosaminyltransferase 7 (GalNAc-T7)	1.57 ± 0.03	<0.001
NM_001005376.1	*PLAUR*	Plasminogen activator, urokinase receptor	1.57 ± 0.10	<0.001
NM_020770.2	*CGN*	Cingulin	1.57 ± 0.32	0.034
NM_181501.1	*ITGA1*	Integrin, alpha 1	1.56 ± 0.32	0.049
NM_017617.3	*NOTCH1*	Neurogenic locus notch homolog protein 1	1.55 ± 0.17	0.021
NM_001060.3	*TBXA2R*	Thromboxane A2 receptor	1.52 ± 0.21	0.038
NM_005560.3	*LAMA5*	Laminin subunit alpha-5	1.5 ± 0.17	0.006
NM_022648.4	*TNS1*	Tensin 1	1.5 ± 0.19	0.011
*B: down-regulated genes*				
NM_000214.2	*JAG1*	Jagged 1	−1.58 ± 0.34	0.035
NM_005795.3	*CALCRL*	Calcitonin receptor-like	−1.62 ± 0.04	0.006
NM_032777.9	*GPR124*	G protein-coupled receptor 124	−1.73 ± 0.13	0.023
NM_003469.3	*SCG2*	Secretogranin II	−2.2 ± 1.13	0.043
NM_031479.3	*INHBE*	Inhibin, beta E	−2.28 ± 0.62	0.016
NM_014333.3	*CADM1*	Cell adhesion molecule 1	−2.42 ± 0.57	0.049
NM_000020.1	*ACVRL1*	Activin A receptor type II-like 1	−2.53 ± 0.40	0.009
NM_004369.3	*COL6A3*	Collagen, type VI, alpha 3	−2.62 ± 0.57	0.004
NM_002010.2	*FGF9*	Fibroblast growth factor 9	−2.88 ± 1.78	0.042
NM_000029.3	*AGT*	Angiotensinogen (serpin peptidase inhibitor, clade A, member 8)	−2.88 ± 0.50	0.035
NM_001147.2	*ANGPT2*	Angiopoietin 2	−8.02 ± 1.44	0.002
NM_000201.2	*ICAM1*	Intercellular adhesion molecule 1	−9.94 ± 1.88	<0.001

Gene expression analysis was performed using the human nCounter® PanCancer Progression Panel (NanoString Technologies, Hamburg, Germany). Only significant (*P* < 0.05) fold changes >−1.5 or > +1.5 fold were considered as differentially expressed in DAPK1 ko clones compared to the wildtype. Transcripts with an RNA count of <5 of all samples were excluded as they were considered as non-expressed. The average of clones (clone 7/6: *n* = 2; clone 10/8: *n* = 3; clone 21/9: *n* = 1) vs. HCT116 (*n* = 3) ± is presented. *P* value was calculated by nSolver software (Nanostring Technologies) using *t*-test. Expression profiling data are available online (GEO accession number: GSE130488)

**Fig. 6:**
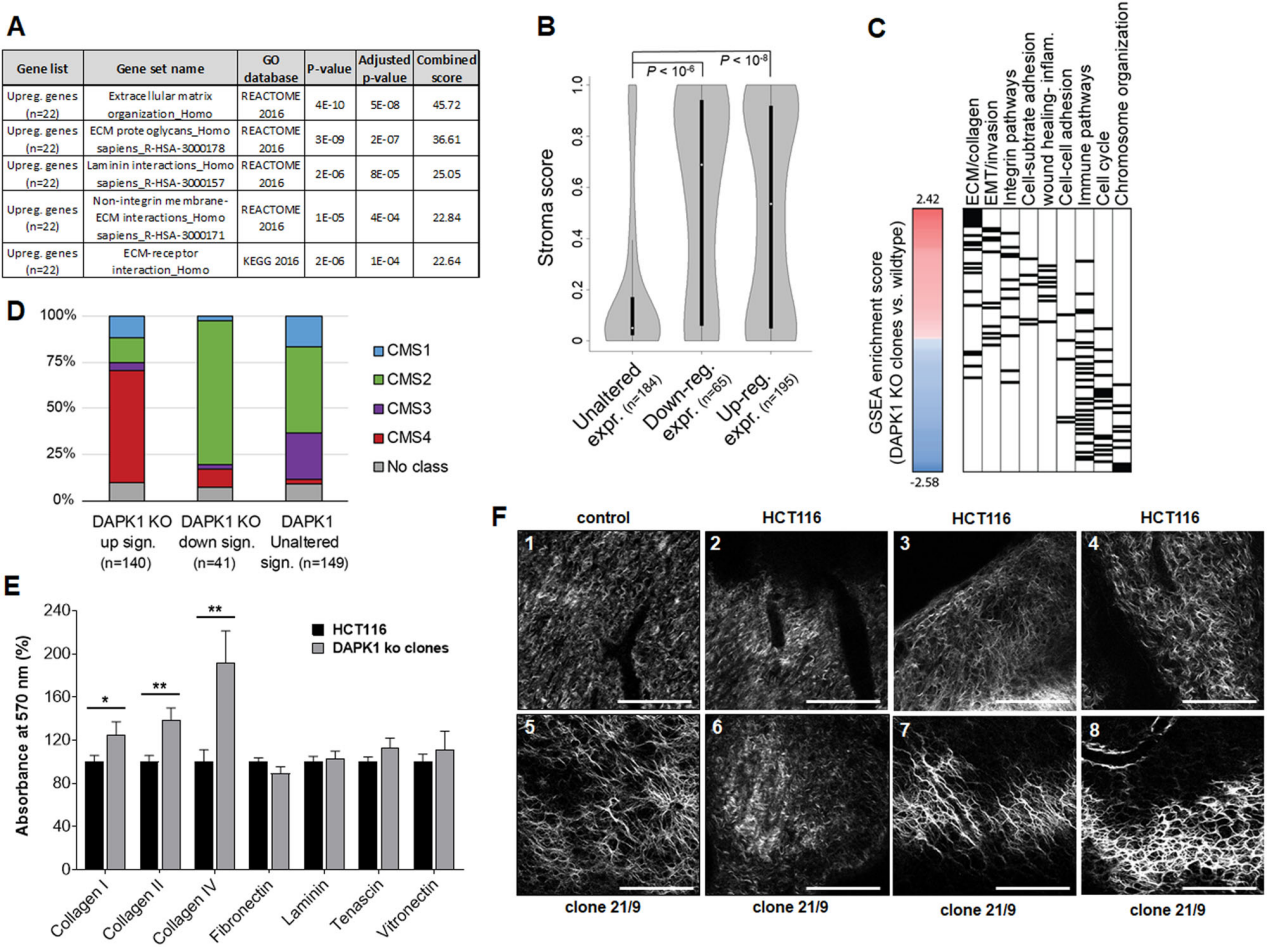
DAPK1 ko clones with stromal expression signature and changed cell–ECM interaction. **a** REACTOME and KEGG 2016-based gene list enrichment analysis of significantly (*P < 0.05; combined score > 20) deregulated genes (*n* = 22 upregulated transcripts) in DAPK1 ko clones (clone 7/6: *n* = 2; clone 10/8: *n* = 3; clone 21/9: *n* = 1) compared to HCT116 cells (*n* = 3) using the Enrichr tool^20^. **b** Violin plots presenting the stroma scores^22^ for transcripts with unaltered (absolute fold change < 1.05), down-regulated (fold change < −1.25) or upregulated (fold change > 1.25) expression in DAPK1 ko clones (clone 7/6: *n* = 2; clone 10/8: *n* = 3; clone 21/9: *n* = 1) compared to HCT116 (*n* = 3). *P* values are indicated (Wilcoxon-rank sum test). **c** Pre-ranked Gene-set Enrichment analysis (GSEA) of fold change values (770 genes) profiled by nCounter® PanCancer Progression Panel (NanoString Technologies) performed using the Molecular Signatures Database v6.2. Gene sets were manually categorized according to gene function. A list of gene-set titles and normalized enrichment scores can be found in Supplemental Table 1). Color code: high normalized gene-set enrichment scores in DAPK1 ko clones (red) and HCT116 (blue). **d** Bar plots presenting the overlap between Consensus Molecular Subtype (CMS) and DAPK1 ko signature classifications in colorectal cancer (CRC) samples (TCGA COREAD cohort). Three categories of CRC samples were defined: DAPK1 ko up, DAPK1 ko down and DAPK1 ko unaltered using the nearest template prediction module^25^. **e** ECM cell adhesion of HCT116 cells and DAPK1 ko clones to collagen I, II and IV, fibronectin, laminin, tenascin and vitronectin. Data are presented as mean ± SD (*n* = 3). Significances were calculated using multiple t-test (Collagen I: *P = 0.049; Collagen II: **P = 0.01; Collagen IV: **P = 0.01). **f** Ex ovo SHG microscopy images showing representative collagen patterns of CAMs harboring (2–4) HCT116 or (5–8) clone 21/9 micro-tumors. A CAM without tumor cells served as naïve control (1). Scale bar = 50 µm.

We inspected deregulated genes to identify biological processes possibly affected by DAPK1 loss in the ko clones. Foremost, gene list enrichment analysis of the 34 significantly deregulated transcripts suggested that they were involved in regulating tumor–stroma interactions, in particular altered ECM organization and matrix adhesion was characteristic for upregulated transcripts (Fig. 6a). In agreement with a role in tumor-stroma modulation, we found that both sets of up- and down-regulated transcripts had a significantly higher “stroma score” (i.e. transcripts primarily expressed by non-epithelial cells within the tumor stroma) than unaffected genes when analyzing all transcripts (Fig. 6b). To closer investigate which tumor–stroma processes were up- and down-regulated upon DAPK1 ko we performed pre-ranked gene-set enrichment analysis (pre-ranked GSEA) using all gene sets in the BROAD Database (MsigDB) gene-set collection v6.2. Handpicked gene sets representing the biological properties that correlate with the DAPK1 ko phenotype in Fig. 6c. We found that gene sets for organization and binding to ECM and mesenchymal transition had higher Normalized Enrichment Scores (NESs) in DAPK1 ko clones, whereas gene sets for immune pathways, cell cycle and chromosome/chromatin organization had negative NESs (Fig. 6c). Collectively, these analyses suggested that loss of DAPK1 in HCT116 cells affected transcripts involved in enhancing tumor–stroma binding and mesenchymal transition. Notably, classification of TCGA CRC samples (COREAD) using NTP suggested that the aggressive CMS4 subtype tumors were indeed associated with the gene signature upregulated in the DAPK1 ko clones as compared to down- or unaffected gene signatures (Fig. 6d).

**Fig. 7:**
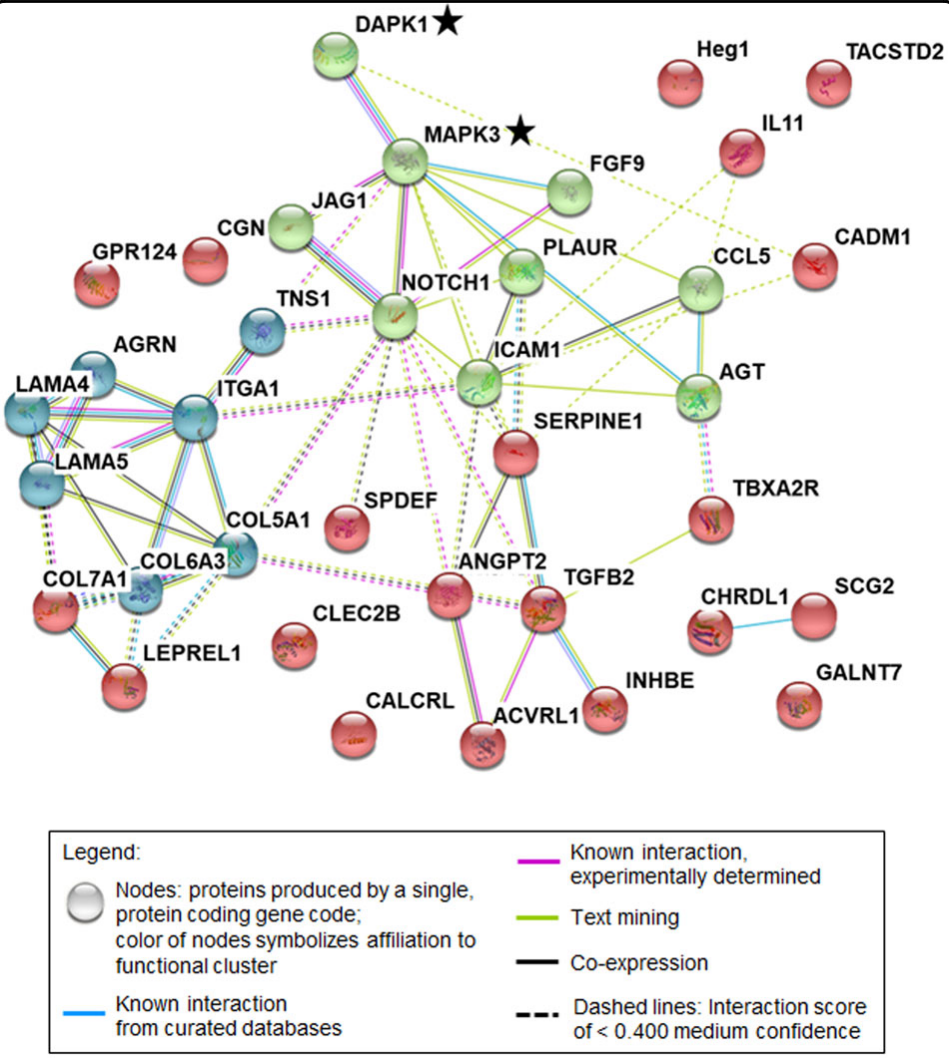
Visualization of suggested DAPK1/MAPK3(ERK2)/ICAM1/TACSTD2 signaling found via STRING database search of top 34 dysregulated genes of DAPK1 ko panel. Black star: additional input to STRING analysis. Color scheme: green: Notch1 cluster, blue: cluster of ECM modulators, red: cluster of more distant interactions. For full gene names and gene reference please refer to Table 1.

**Fig. 8:**
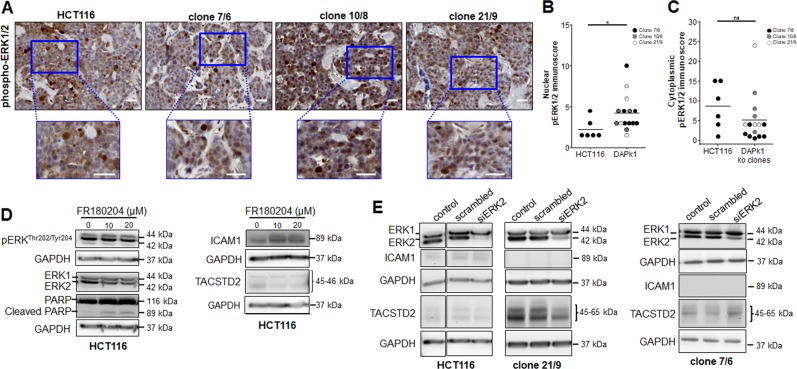
ERK mediated signaling in DAPK ko clones. **a** Representative images of phospho-ERK1/2 expression in CAM micro-tumor paraffin sections by immunohistochemistry. Blue boxes: ×2 zoomed in; scale bar = 50 µm. **(b)** Nuclear and **(c)** cytoplasmic immunoscore of phospho-ERK1/2 expression in CAM micro-tumors obtained by multiplying staining intensity with percentage positivity divided by 10 (HCT116: *n* = 6; clone 7/6: *n* = 7; clone 10/8: *n* = 4; clone 21/9: *n* = 4). A Mann–Whitney test was performed to calculate significance between two groups (*P = 0.021; ns *P* = 0.1539). **d** Western Blot analysis of pERK^Thr202/Tyr204^, ERK1/2, PARP and cleaved PARP, ICAM1 and TACSTD2 protein expression in HCT116 cells treated with 0, 10, and 20 µM of ERK1/2 inhibitor (FR180204) for 48 h. Representative images are shown. GAPDH served as loading control. **e** Effects of siERK2 in HCT116, clone 21/9 and clone 7/6 cells on ERK/1/2, ICAM1 and TACSTD2 protein levels were investigated by Western Blot analysis in comparison to the corresponding non-treated and scrambled-treated cells. Cells were harvested 48 h after transfection. Representative images of two independent experiments are shown. GAPDH was used as loading control.

To support the suggestion that ECM interaction was altered in DAPK1 ko clones we performed an ECM cell adhesion array, which revealed that DAPK1 ko cells significantly benefit from loss of DAPK1 regarding binding to collagen I, II and IV (*P < 0.05, **P < 0.01; **P < 0.01; Fig. 6e). Indeed, SHG images of the surrounding CAM showed differences in collagen alignment for HCT116 and DAPK1 ko tumors. Whereas there were areas of very similar collagen alignment as found in the control CAM without xenografts (Fig. 6f—1 versus 2,6), DAPK1 ko engrafted CAMs showed a higher variation in the extend and arrangement of collagen fibers. DAPK1 ko was majorly associated with a more regular and polarized collagen fiber structure (Fig. 6f—7,8), abundant thicker bundles (Fig. 6f—5,7,8) with network building (Fig. 6f—5,7,8). In CAMs engrafted with HCT tumors we could hardly detect any aligned fibers in the matrix and the fibers were oriented in a multitude of directions (Fig. 6f —2–4).

### DAPK1/ERK2 signaling axis targets TACSTD2 expression

It has been reported that ERK when phosphorylating DAPK1 at Ser^735^ induces a negative feedback loop since DAPK1´s death domain is then binding to ERK, blocking its nuclear translocation and finally triggering apoptosis induction^31^. To analyze oncogenic players of the DAPK1-ERK2 mediated regulation axis, we performed a STRING analysis including the 34 deregulated DAPK1-dependent genes (Table 1) and added DAPK1 and ERK2 (MAPK3) into this network (Fig. 7). Indeed, ERK2 was situated in close neighborhood of DAPK1 (Fig. 7). The network itself consisted of two major nodules: NOTCH1 and ECM modulators. Interestingly, DAPK1 seems to regulate the network from outside linking the two clusters. Notably, so far, CLEC2B, Heg1, GALNT7, CALCRL, GPR124, CGN, and TACSTD2 have not been studied for their association with the DAPK1 network. The DAPK dependent gene signature involved six different dysregulated ECM components (Fig. 7).

As expected, pERK1/2 protein was mainly localized in the nuclei when evaluating CAM xenografts of DAPK1 ko clones (Fig. 8a–c). Inhibition of ERK1/2 kinase activity using 10 and 20 µM of ERK1/2 inhibitor (FR180204) in HCT116 slightly increased cleaved PARP and ICAM1 levels and decreased TACSTD2 levels (Fig. 8d). Next, we inhibited ERK2 signaling by siERK2 transfection of HCT116, clone 21/9 and 7/6 cells (Fig. 8e). An ERK2 inhibition Western Blot analysis showed decreased TACSTD2 levels in HCT116 cells and in 21/9 cells whereas ICAM1 remained undetectable (Fig. 8e) suggesting that DAPK1-ERK2 axis might be partly involved in regulation of TACSTD2, but not in regulation of ICAM1.

## Discussion

In this study, we firstly report a novel DAPK1-mediated network associated with metastatic potential of colorectal tumors. We show that under DAPK1 loss, colon tumor cells gain ability to modulate the ECM consequently increasing their metastatic potential in vitro and in vivo. We hypothesize that this hallmark of cancer could be partly driven by the DAPK1-ERK axis.

So far, there have been only a few hints obtained from CRC patient data suggesting that DAPK1 functionally acts as a metastasis suppressor in the colonic epithelium. It has been shown in two independent studies that DAPK1 expression was decreased in tumors that had already metastasized at time of diagnosis^3,4^. In papillary thyroid cancer, pituitary tumors, hepatocellular, and esophageal carcinoma DAPK1 promoter hypermethylation was correlated with advanced tumor stages and worse prognosis^32–35^. The molecular basis of these observations has only been little understood.

Here, we give novel experimental evidence for DAPK1´s role in migration, invasion, and ECM modulation and antimetastatic potential in CRC cells. DAPK1 seems to exert sensor functions under conditions where an interaction with ECM and basement membrane components is possible. Our study required a long-term and stable gene knockout which was implemented by the CRISPR/Cas9 technology. Slight differences in molecular and functional readouts among the randomly picked DAPK1 ko clones are caused by the monoclonal strategy which was applied to the heterogeneous HCT116 parental cell line. Thus, as a result of mixed or inefficient gene editing (a per-cell rate of 30–60% CRISPR ko is reported), an efficient conversion to an uniform cell population which was required for proper gene ko assessment, can be reached only by single-cell cloning^36^. Although a complete loss of DAPK1 expression was verified, we showed that the genetic background of single clones was varying especially regarding stem cell marker expression. Such genetic background pattern might modify the DAPK1 signature, however, can be mostly neglected in our approach since we only focused on genes that were commonly deregulated in all three DAPK1 clones.

We identified a DAPK1-driven network of 22 genes upregulated under loss of DAPK1 in HCT116 cells. Literature research (Pubmed, September 2019) revealed that 73% (IL11, TACSTD2, CLEC2B, LAMA4, CCL5, SERPINE1, TGFB2, COL7A1, GALNT7, PLAUR, CGN, ITGA1, NOTCH1, TBXA2R, LAMA5, TNS1) of upregulated genes found in our study have already been described in previous studies in CRC. In particular, IL11 promotes growth of neoplastic epithelium^37^, and is associated with poor differentiation, a large tumor size, lymph node metastasis and overall low survival of CRC patients^38^. CLEC2B belongs to C-type lectins which facilitate the tumor metastasis in many cancers^39^. Interestingly, two laminins LAMA4 and LAMA5 were upregulated in DAPK1 ko signature^40,41^. LAMA5 is required for growth of hepatic metastases where it promotes branching angiogenesis and regulates Notch signaling^42^. In accordance with the observed increased collagen binding in vitro and in vivo in DAPK1 ko clones, ITGA1, which encodes the alpha 1 subunit of integrin receptors (collagen surface receptor), has been found to be upregulated in the DAPK1 ko clones. Moreover, the negative regulator of the MMP-associated proteolytic network, the serine protease inhibitor SERPINE1 (plasminogen activator inhibitor-1) seemed to be associated with aggressive tumor behavior in CRC^43,44^. It was reported to maintain an angiogenic “scaffold” and stabilizes nascent capillary structure which is in agreement with the observed pro-angiogenic phenotype under DAPK1 loss.

For 23% (P3H2, CHRDL1, COL5A1, HEG1, AGRN) DAPK1 dysregulated genes their role in CRC is completely unknown. Taken together, the identified overall gene expression signature in the DAPK1 ko cell lines confirms our hypothesis, that DAPK1 might inhibit CRC progression and metastasis.

Then we focused further on TACSTD2 (TROP2), a cell surface receptor that transduces Ca^2+^ signals. Although TACSTD2 was found to be highly expressed in many cancer types, only a few very recent reports are available about its functional role in cancer^45^. TACSTD2 has been described as an oncogene promoting cell proliferation, epithelial-to-mesenchymal transition, and metastasis in bladder and colon cancer^46,47^. EpCAM positive circulating breast cancer cells were enriched for TACSTD2 expression linking it to a higher metastatic capability of tumor cells^48^. In our study, we showed in vitro and in vivo that DAPK1 loss led to higher TACSTD2 expression in an ERK-dependent manner. Thus, one of the tumor suppressor functions of DAPK1 might be partly mediated by suppressing the metastasis associated TACSDT2. Loss of DAPK1 in tumor buds at tumor invasion front of CRC as we previously described^4^ would consequently add more aggressive properties to disseminating tumor cells. This hypothesis is supported by findings that TACSTD2 promotes cell motility in prostate cancer cells by modulating the ß1 integrin signaling and increases wound healing by promoting stem cell survival^49,50^. DAPK1 is known to inactivate integrin β1 dependent matrix survival signals and TACSTD2 would potentiate this signaling, in a DAPK1 loss situation also further reducing the integrin-dependent ICAM1 signaling. Indeed we found an up-regulation of integrin β1 expression (1.22-fold, *P* = 0.07) when DAPK1 is lost. During metastatic cascade, tumor cells interact not only with several immune cells from the tumor environment, but also with fibronectin, laminin, and type I collagen of the basement membrane. In our study, we showed for the first time that the loss of DAPK1 was associated with a higher collagen binding. The reorganized collagen fiber meshwork of the CAM around DAPK1 ko xenografts seems to trigger cell invasion and distant metastasis formation. This might also explain the better and faster adaptation of DAPK1 loss tumor cells in the physiological environment of PCTS experiment. DAPK1 loss might promote dissemination seen as increase in number of tumor buds and remodeling of the CAM collagen matrix triggering the colonization of tumor cells at a secondary site since we found significantly more metastasis in chicken embryonic organs from DAPK1 ko xenografts.

Tumor progression with metastasis is associated with severe alterations in cell–cell and cell–matrix interactions. One of the genes remarkably deregulated under DAPK loss was found to be the cell surface protein ICAM1. Here we describe a role for DAPK in regulating ICAM1 and subsequently in interaction with ECM components of the tumor microenvironment. Indeed, we showed that the loss of DAPK1 and ICAM1 was associated with an up-regulation of integrins that are specifically interacting with collagens. Since under DAPK1 loss both ICAM1 transcripts and ICAM1 protein are completely lost we believe in a transcriptional regulation mechanism. ICAM1 has been shown mainly to act in different cell types as a metastasis suppressor^51–53^. A lower number of ICAM-1 positive cells has been observed in metastasizing CRC^54^. Under conditions where ICAM1 is lost, the tumor microenvironment is remarkably rebuilt and an increased M2 polarization of macrophages has been observed^55^ leading to maintenance of acute inflammation and an attenuated tissue repair^56^. There is still controversy about the role of ICAM1 in tumor metastasis since high ICAM1 expression has been described in advanced melanoma^57^. So ICAM1 might fulfill different functional properties dependent on the cellular subtype and the specific pathologic situation. DAPK1 has been shown to be inactivated by Netrin-1^58^ to potentiate tumor-associated vessel formation in metastatic lung cancer^59^. Through modulating the tumor-associated vasculature DAPK might also be involved in shaping the tumor microenvironment.

We have identified a connection between DAPK1, ICAM1 and MAP kinase ERK1/2 in STRING analysis. DAPK1 is a phosphorylation substrate of ERK and vice versa. When DAPK1 is phosphorylated by ERK at Ser735 (interacting via its death domain) it holds ERK in the cytoplasm^31^, thus preventing its nuclear translocation. This reciprocal regulation leads to a positive feedback loop that promotes apoptosis. Thus, we speculate that endogenous DAPK1 loss could lead to an increase in nuclear shuttling of active pERK1/2. Indeed we observed more pERK1/2 protein in the nucleus in 2D immunofluorescence, in cell fractionation, and in vivo in CAM xenografts of DAPK1 ko clones. Since ERK has to translocate into the nucleus to regulate gene transcription, cell proliferation and differentiation^60^, we suggest that DAPK1 loss at the invasion front of CRC should have to do at least in part with an ERK1/2 triggered signaling cascade. Since inhibition of ERK signaling did not affect ICAM1 expression level we suggest that ERK signaling is not majorly involved in DAPK1-mediated ICAM1 expression.

In summary, we have successfully modeled the in vivo situation that DAPK1 is mostly lost at the tumor invasion front of CRC. We suggest a novel and even more comprehensive picture of DAPK1´s antimetastatic functions by giving the first time experimental data that it diminishes an effective tumor cell–ECM interaction. DAPK1 exerts its tumor suppressor function at least partly via suppressing the metastasis associated TACSDT2. The potential of the DAPK1 loss gene signature for metastasis prediction and therapy resistance should be further investigated.

## Supplementary information

Figure S1

Figure S2

Figure S3

Figure S4

Figure S5

Table S1
